# Decompressive Hemicraniectomy After Cerebral Fat Embolism

**DOI:** 10.7759/cureus.61338

**Published:** 2024-05-29

**Authors:** Zachary Sokol, Glenn A. A Gonzalez, Alejandro Lopez, Jim Harrop, Pascal Jabbour

**Affiliations:** 1 Lewis Katz School of Medicine, Temple University, Philadelphia, USA; 2 Neurological Surgery, Thomas Jefferson University, Philadelphia, USA; 3 Neurosurgery, Thomas Jefferson University Hospital, Philadelphia, USA; 4 Neurological Surgery, Thomas Jefferson University Hospital, Philadelphia, USA; 5 Neurosurgery, Thomas Jefferson University, Philadelphia, USA

**Keywords:** case report, cerebral fat embolism, fat embolism, intracranial pressure, decompressive hemicraniectomy

## Abstract

Fat embolism syndrome is a common occurrence after orthopedic trauma and surgery. Cerebral fat embolism (CFE) may arise after fat globules enter the arterial circulation. The neurological manifestations of CFE vary and generally carries a favorable outcome. A small number of reports exist regarding patients with CFE who experienced severe neurological deficits and significant edema on radiographic studies, and subsequently underwent decompressive hemicraniectomy (DHC), some of which had full neurological recoveries. Here, we present the case of a 21-year-old male who presented after a motorcycle accident with multiple orthopedic injuries, who after fixation did not awake from anesthesia. The patient was ultimately found to have cerebral fat emboli, and developed significant edema and swelling. The patient underwent DHC with subsequent cranioplasty and returned to his neurological baseline seven months after his initial injury. DHC for CFE has been described in a few cases with some patients have had substantive recoveries, including the present case. This case emphasizes the importance of promptly recognizing and reversing elevated intracranial pressures and the possibility of promising recoveries.

## Introduction

Fat embolism syndrome (FES) is a common finding after orthopedic trauma. It remains a relatively ill-defined clinical entity with potential impacts on many organs. Fat embolism syndrome commonly occurs after long bone fractures, and can be exacerbated by orthopedic fixation. The rate at which FES occurs varies widely based on the diagnostic criteria used, and the initial injury. The etiology of damage is attributed to occlusion of capillaries by microemboli, and hypoxemia has been suggested as a marker for subclinical FES. All organs may be impacted by FES, however, the most severe forms involve emboli to the brain, lungs, and heart [1,2].

Fat embolization to the brain of clinical significance is relatively uncommon, comprising approximately 1% of FES. Classically it causes occlusion of microvasculature leading to a “starry sky” infarct pattern on MRI. These cases are typically managed conservatively. The mechanism by which emboli enter the brain is thought to be often due to a patent foramen ovale (PFO) allowing fat emboli to enter the cerebral circulation while bypassing the pulmonary circulation, although a majority of patients with cerebral fat emboli (CFE) do not have a PFO, suggesting other mechanisms. Large vessel occlusion (LVO) due to fat embolism syndrome has been reported. Neurological decline typically occurs hours after initial insult, and initial non contrast head CT is often unrevealing [3-6].

Here we report the case of a 21-year-old male who, after significant orthopedic trauma, did not awake from anesthesia immediately post-procedure. In the following days, he was found to have significant fat emboli to the brain, with subsequent edema and herniation. He was taken for decompressive hemicraniectomy (DHC) and subsequently had a full neurological recovery. 

## Case presentation

A 21-year-old male with no significant past medical history presented as a trauma alert following a motorcycle accident. He had a Glasgow Coma Scale (GCS) score of 15, stable vital signs, and exhibited no neurological deficits. Orthopedic injuries included a nondisplaced fracture of the left femoral neck, a displaced transverse fracture of the left femoral shaft, an open displaced transverse fracture of the left tibia, and an open displaced oblique fracture of the left fibular shaft.

**Figure 1:**
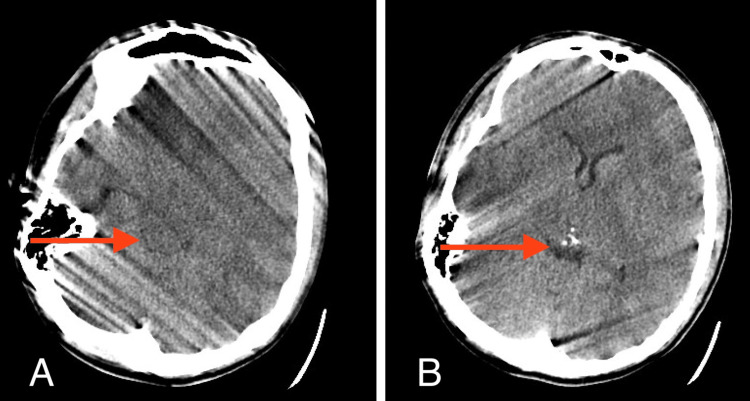
Axial head CT (A, B), obtained immediately after orthopedic fixation showing no abnormalities and open cisterns (red arrows)

**Figure 2:**
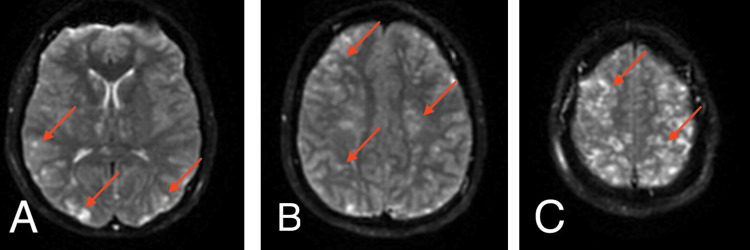
Axial DWI MRI (A, B, C), red arrows demonstrating "starry sky" infarcts, most consistent with CFE. DWI: Diffusion-weighted imaging; CFE: cerebral fat embolism

The patient underwent surgical fixation of the injuries to his left femur, tibia, and fibula. The surgery proceeded without any abnormal intraoperative events; however, the patient did not regain consciousness post-anesthesia. Despite the administration of reversal agents and the observation of four twitches in response to electrical stimulation, the patient remained unresponsive with a GCS score of 3T. Laboratory tests were unremarkable, except for an elevated lactate level of 7.9. An urgent head CT was performed, which showed no abnormalities (Figure 1). The patient was transferred to the ICU, where his examination remained GCS 3T off sedation. Continuous EEG revealed no seizure-like activity. An MRI showed diffuse bilateral infarcts, raising concerns for a fat embolism (Figure 2). Subsequently, on post-operative day (POD) 2, the patient was transferred to a quaternary care hospital with a neurological critical care unit.

**Figure 3:**
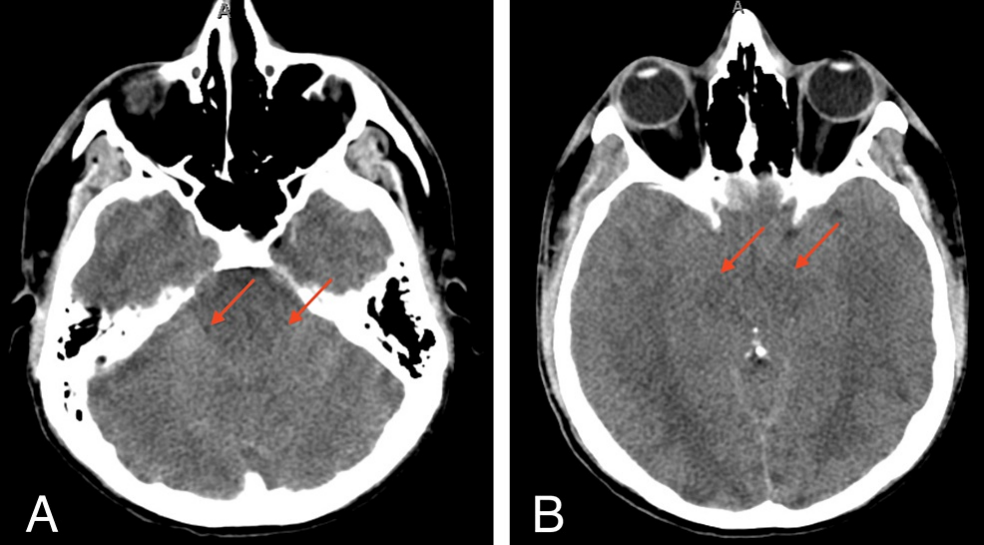
Axial head CT demonstrating diffuse cerebral edema and effacement of basal cisterns (red arrows), most consistent with severe edema and herniation

**Figure 4:**
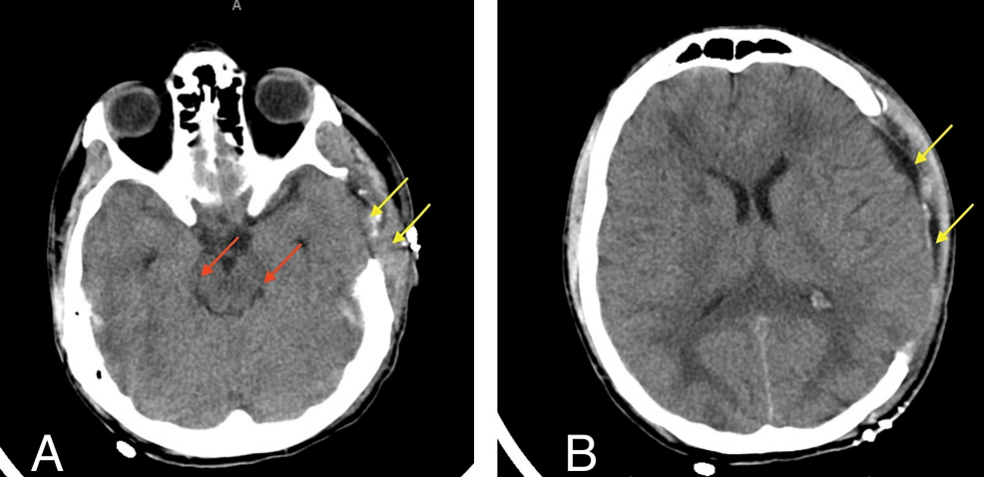
Axial Head CT after DHC demonstrating adequate decompression of cisterns (red arrows), and craniectomy (yellow arrows) DHC: decompressive hemicraniectomy

Upon arrival, his examination was GCS 9 (E3M5V1T); he opened his eyes to stimulation, had reactive pupils, and protective reflexes were present. He exhibited localization with his bilateral upper extremities and withdrawal with his bilateral lower extremities. Approximately 12 hours later, the patient's neurological examination worsened, with nonreactive pupils and extensor posturing. An emergent head CT scan indicated diffuse cerebral edema and effacement of the basal cisterns, consistent with herniation (Figure 3). He underwent an emergent decompressive hemicraniectomy (DHC) and placement of an intracranial pressure (ICP) monitor (Figure 4). Initial ICP readings ranged from 9-11 cm H2O after decompression. The patient was maximally sedated immediately post-operatively in anticipation of maximal swelling during this period.

The patient's ICP remained well controlled, and his examination remained stable with reactive pupils and present protective reflexes, although he still could not follow commands. On POD 36, he was transferred to a rehabilitation facility. In rehab, he followed commands, and exhibited 2/5 strength in his right upper extremity (RUE) and 3/5 strength in his left upper extremity (LUE), with spontaneous eye opening.

**Figure 5:**
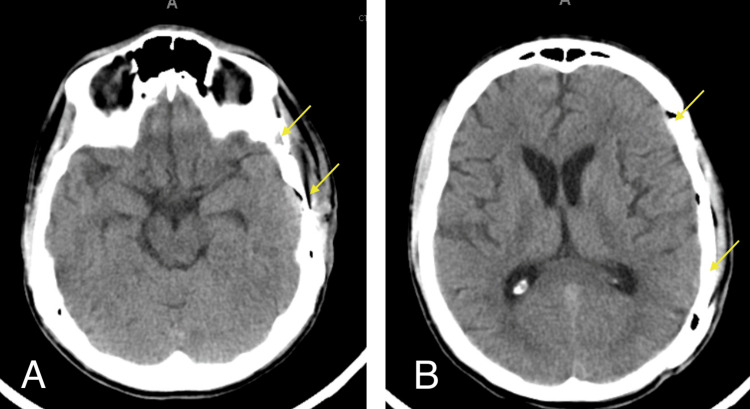
Axial head CT after cranioplasty (yellow arrows)

Three months post-surgery, he regained the ability to follow commands and speak, though he was wheelchair-bound (modified Rankin Score 3). His bone flap (which had been preserved cryogenically) was replaced shortly thereafter (Figure 5). Four months after the cranioplasty, and seven months after his initial injury, he returned to his neurological baseline before the accident, exhibiting no focal deficits except for mild expressive aphasia (modified Rankin Score 0).

## Discussion

The rate at which FES occurs varies widely based on the diagnostic criteria used, and the initial injury. The etiology of damage is attributed to the occlusion of capillaries by microemboli, and hypoxemia has been suggested as a marker for subclinical FES. While all organs may be impacted by FES, the most severe forms involve emboli in the brain, lungs, and heart [1,2].

Diagnosis of fat embolism is a challenging clinical scenario. Rates of fat embolism vary widely based on these criteria, from <1% to 88% in patients undergoing orthopedic surgery. Disruption of the fat in marrow-containing-bones passes into venous sinuses and subsequently into systemic circulation. Some authors have posited that simple vascular occlusion does not account for the pathophysiology of FES. Additional biochemical pathways for injury have been described, including inflammatory responses to free fatty acids, leading to effects on the brain and other organ systems, including coagulopathies and shock states [1-4].

CFE arises after fat emboli enter the arterial circulation. Two pathways have been proposed whereby fat globules enter the arterial circulation: through a patent foramen ovale, and through the passage of microglobules through the pulmonary circulation. CFEs comprise 1-10% of FES cases and typically do not lead to such severe neurological presentations. Typically, when deficits do arise, they occur hours after orthopedic fixation, and symptoms gradually worsen after 24-72 hours. Typically, fat emboli enter the cerebral microcirculation leading to the characteristic “starry sky” pattern on MRI, however large vessel occlusion has also been described, and large vessel occlusion from fat embolism seems to carry a worse prognosis than CFE [1,2,4,6].

The presentation of CFE may vary from mild confusion to coma, along with seizures and focal deficits. The diagnosis is made clinically, and some imaging studies may be of utility, especially DWI MRI, which may show scattered foci of diffusion restriction. Treatment is generally supportive, including airway protection. Corticosteroids have been studied, and the results have been variable. Early fixation of fractures (within 24 hours) has been shown to reduce rates of FES. CFE typically carries a favorable outcome, with mortality ranging from 5-15%, with the majority of these attributable to respiratory failure. Small studies have evaluated long-term cognitive outcomes in patients with CFE and have found that younger patients typically fare well in the long term [3,5-9].

This case report underscores the importance of rapid diagnosis and treatment for FES. DHC has been well-established as a treatment in cases of severe TBI and severe ischemic stroke [9]. However, some reports have described patients with CFE who, after undergoing DHC, experienced poor outcomes (Table 1) [5,7]. Notably, one case involved a 15-year-old female who initially presented postoperatively with a GCS score of 11 and subsequently lost brainstem reflexes several days later, necessitating DHC. Despite these challenges, she ultimately had a favorable outcome [10]. This case suggests that the initial neurological injury from the fat embolism may not be permanent, and rapid decompression may be warranted in situations involving a significant stroke burden.

**Table 1:** Summary of previously reported cases of DHC after CFE DHC: decompressive hemicraniectomy; CFE: cerebral fat embolism

Case	Clinical Presentation	Outcome
Ooi et al., 2021 [5]	69-year-old female with femoral fracture after mechanical fall. Initially GCS 15, deteriorated to GCS 8 immediately after femur fixation. The following day, patient was found to have significant cerebral edema, underwent DHC.	No meaningful neurological improvement, palliative measures pursued, and patient died in hospital.
Kellogg et al. 2013 [7]	58-year-old female presented with femur fracture after mechanical fall, initially GCS 15. Patient developed tonic-clonic seizure, did not return to neurological baseline. Significant swelling on day 3, underwent DHC.	Modified Rankin Score of 4 at 6 months.
Couturier et al., 2019 [10]	15-year-old female with femur fracture secondary to trauma. Initially GCS 15, deteriorated to GCS 11 before fixation, underwent femoral nailing with intraoperative episode of hypotension. Presented with fixed and dilated pupils 24 hrs after initial injury, underwent DHC at that time.	Full neurological recovery within 6 months, with occasional headaches.
Present case	21-year-old male with multiple fractures, became GCS 3 immediately after ORIF, developed significant brain edema with herniation and underwent DHC at that time.	Full neurological recovery within 6 months, some slow speech

## Conclusions

FES after orthopedic trauma is a well-established clinical entity. CFE is less common. Treatment in both generally consists of supportive care. A small number of case reports have detailed surgical intervention (DHC) after CFE for patients with severe cerebral edema refractory to other measures. This case underscores the importance of promptly reversing intracranial pressure and optimizing cerebral perfusion, as some instances have resulted in very promising recoveries.
